# Prevalence and direct health cost of mental diseases in Hungary - analysis of the National Health Insurance Fund’s data

**DOI:** 10.1192/j.eurpsy.2022.869

**Published:** 2022-09-01

**Authors:** P. Dr. Fadgyas-Freyler

**Affiliations:** Corvinus University Budapest, Health Economics, Budapest, Hungary

**Keywords:** mental health patient numbers, mental health costs, direct mental health care spending, mental care costs Hungary

## Abstract

**Introduction:**

According to international publications the burden of mental diseases is considered to be significant and rising.

**Objectives:**

Scope of analysis is to present 1) patient numbers and 2) direct mental health costs from the database of the National Health Insurance Fund Hungary for patients with F00-F99 ICD code between 2015-2019.

**Methods:**

An Oracle database was created with direct mental care costs for each patient in a given year with a three-digit ICD code and type of care (primary, specialist, prescribing) and handled via sql queries. Data on capacity and performance came from the NHIF and NSO website for 2008-2019 and were handled via Microsoft Excel.

**Results:**

Mental problems affected 3 million people (more than 30% of the population) in a five year period, though patient numbers are continuously declining. Almost half of the patients only visit a general practitioner and don’t get a prescription. There is also a drop in proportional mental spending which has fallen from 5,03% to 4,02%. This tendency is accordance with international findings. There is a dramatic fall of inpatient cases and a growing number of outpatient interventions, though we see a move from individual therapy sessions to group interventions and a decline in specialist psychotherapy sessions. We can see a shift towards more young patients both in inpatient and outpatient setting.

**Conclusions:**

The analysis raises the question whether declining patient numbers and shrinking proportional spending are due to smaller provider capacities and unmet need or a mentally healthier population.

**Disclosure:**

No significant relationships.

